# Intrinsic Regulatory Role of RNA Structural Arrangement in Alternative Splicing Control

**DOI:** 10.3390/ijms21145161

**Published:** 2020-07-21

**Authors:** Katarzyna Taylor, Krzysztof Sobczak

**Affiliations:** Department of Gene Expression, Institute of Molecular Biology and Biotechnology, Faculty of Biology, Adam Mickiewicz University, 61-614 Poznan, Poland; kksiazek@amu.edu.pl

**Keywords:** RNA structural arrangement, alternative splicing, RNA structure modulators, RNA structure- and splicing-associated diseases

## Abstract

Alternative splicing is a highly sophisticated process, playing a significant role in posttranscriptional gene expression and underlying the diversity and complexity of organisms. Its regulation is multilayered, including an intrinsic role of RNA structural arrangement which undergoes time- and tissue-specific alterations. In this review, we describe the principles of RNA structural arrangement and briefly decipher its *cis*- and *trans*-acting cellular modulators which serve as crucial determinants of biological functionality of the RNA structure. Subsequently, we engage in a discussion about the RNA structure-mediated mechanisms of alternative splicing regulation. On one hand, the impairment of formation of optimal RNA structures may have critical consequences for the splicing outcome and further contribute to understanding the pathomechanism of severe disorders. On the other hand, the structural aspects of RNA became significant features taken into consideration in the endeavor of finding potential therapeutic treatments. Both aspects have been addressed by us emphasizing the importance of ongoing studies in both fields.

## 1. Introduction

Splicing is an essential process of gene expression in eukaryotes, leading to the production of mature RNA species including messenger RNAs (mRNAs) [1], long non-coding RNAs [2], and transfer RNAs [3]. Nearly 95% of protein encoding genes in eukaryotes undergo alternative splicing (AS) in which exonic regions, either entire exons or their parts, are alternatively removed or introns are retained giving rise to diversified variants of proteins. A single precursor mRNA (pre-mRNA) may be a substrate of alternative splicing, generating multiple protein isoforms which carry differential properties encoded by alternative exons. For example, pre-mRNA of Muscleblind-like 1 (*MBNL1*) splicing factor is spliced to several mRNA isoforms and the majority of them encode the MBNL1 protein isoforms of distinct cellular localization, splicing activity, stability, and propensity for dimerization [4]. Moreover, AS determines proteins’ production rates and their half-life via multiple downstream processes including nonsense-mediated RNA decay (NMD) or non-stop decay [5,6]. Interestingly, aforementioned MBNL1 determines NMD of Chloride channel protein 1 (*CLCN1*) pre-mRNA by regulating splicing of exon 7a which carries an in-frame premature termination codon (PTC) [7]. MBNL1-mediated exclusion of exon 7a leads to the production of a functional skeletal muscle specific chloride channel. However, disease-associated reduction of functional pool of MBNL1 results in exon 7a inclusion and NMD-based turnover of *CLCN1* transcript which manifests in reduced chloride ion conduction and myotonia affecting skeletal muscles in myotonic dystrophy type 1 (DM1) [8]. 

**Figure 1:**
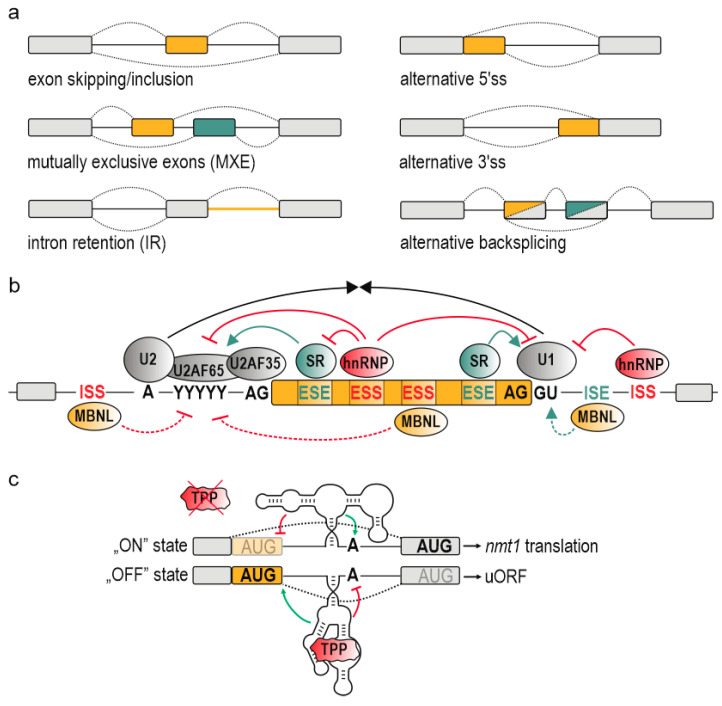
Schematic presentation of types of alternative splicing and its general regulation. (**a**) Different types of alternative splicing including alternatively spliced exons, introns, 5′ss and 3′ss. Alternative exons are marked in orange and green; constitutive exons are marked in grey. (**b**) Simplified scheme of alternative exon definition by components of spliceosome (marked in grey), auxiliary *cis*-acting elements (ESS, ESE, ISE, ESS, A, YYYYY) and *trans*-acting protein factors (SR, hnRNP, MBNL). Detail description is included in the main text. Alternative exon is marked in orange; SR, serine/arginine rich proteins; hnRNP, heterogenous nuclear ribonucleoprotein; MBNL, Muscleblind-like protein; ESE and ESS, exonic splicing enhancer and silencer, respectively; ISE and ISS, intronic splicing enhancer and silencer, respectively; A, branch point; YYYYY, Py-tract; green arrows, positive splicing regulation; red arrows, negative splicing regulation; black arrows, reciprocal relation of spliceosome components for exon definition. (**c**) Fungal riboswitch within intron 1 of N-myristoyltransferase 1 (*nmt1*) gene [52]. It base pairs with alternative 5′ss enabling the selection of an upstream 5′ss and production of a functional nmt1 protein (“ON” state). Under excess of thiamine pyrophosphate (TPP) ligand, the TPP recognizes and binds to this RNA element imposing its structural alterations as well as rearranging the accessibility of adjacent *cis*-acting elements. In consequence, alternative 5′ss and upstream translation initiation codon are selected leading to reduction of *nmt1* expression (“OFF” state). uORF, upstream open reading frame.

AS events can be arranged into the following basic categories: exon skipping, mutually exclusive splicing, intron retention, and selection of alternative 5′ and 3′ splice sites (ss) [5] (Figure 1a). It is worth mentioning alternative backsplicing as a unique type of splicing, which engages distinct splice donors (5′ss) and upstream splice acceptors (3′ss), leading to the formation of circular RNAs (circRNAs) composed of alternative or constitutive exons with covalently linked ends [9,10,11] (Figure 1a). AS comprises a mediatory pathway for executing important responses to cellular and environmental signals. It enables a proper organism development and an appropriate response to environmental stimuli including heat stress, UV exposure or infections [12,13,14,15,16,17], whereas its impairment underlies a broad range of diseases including cancer, hereditary disorders, and metabolic conditions [18]. The mechanism of AS requires a fine-tuned activity of multiple *cis*-acting elements and *trans*-acting factors, the availability and activity of which may vary at different developmental stages and between tissues [19]. The spliceosome, a large ribonucleoprotein-complex formed by more than 170 proteins and small nuclear RNAs (U1, U2, U4, U5, and U6), constitutes a core of splicing machine which assembles around 5′ and 3′ss and excises introns [20,21,22]. In vertebras, due to short exons and long introns, the spliceosome frequently forms across exons, so called exon definition [23]. In lower eukaryotes, however, the splicing machinery more often defines short introns which flank longer exons, so called intron definition. The recognition of splice sites by spliceosome is determined by combinatorial contribution of multiple features including the strength and structural context of 5′ and 3′ss, polypyrimidine tract (Py-tract), branch point and the presence of auxiliary regulatory signals reflected by *cis*-acting exonic and intronic splicing silencers and enhancers (ESS, ISS, ESE, ISE) [22,24,25,26,27] (Figure 1b). The latter predominantly interact with two ubiquitous families of factors coordinating RNA processing including constitutive splicing and AS; serine-arginine rich (SR) proteins which mainly enhance alternative exon inclusion and heterogeneous nuclear ribonucleoproteins (hnRNPs) which counteract them, although with many exceptions from their primary function as SR proteins may occur as silencers and hnRNPs as enhancers of AS [24,28,29]. Moreover, these *cis*-acting silencers and enhancers comprise an interaction platform for auxiliary *trans*-acting factors recognizing strictly defined or degenerated motifs and represented by various families of RNA-binding proteins (RBPs) whose expression is spatially and developmentally regulated [30,31,32]. An excellent example of this is MBNL1 which promotes adult-like direction of AS of hundreds of transcripts by interaction with 5′-YGCY-3′ motifs (Y stands for a pyrimidine) in pre-mRNA [33]. MBNL1 expression is low in prenatal stages and increases during development becoming a crucial alternative splicing factor mainly in adult muscles, brain, and heart [34,35]. Additionally, MBNL1 may function to repress or activate splicing in a position-dependent manner [36]. Its association with downstream intronic *cis*-acting sites promotes the alternative exon inclusion, whereas the exclusion occurs due to MBNL1 binding within an alternative exon and/or upstream intron (Figure 1b). Reminiscent position-dependent activity characterizes other splicing factors including RNA binding FOX-1 homolog (RBFOX1) [37] and polypyrimidine tract binding protein 1 (PTBP1) [38]. 

In addition to the RNA primary structure (nucleotide sequence), the RNA secondary and tertiary conformation emerged as a source of functionality and a significant layer in AS regulation [39,40,41,42,43,44]. Riboswitches are a leading example of such RNA structures playing a role of gene expression regulators. Riboswitches constitute a specialized class of RNA elements undergoing a dynamic, ligand-induced structural rearrangement which further imposes the alternative RNA folding of adjacent regions and regulates the expression of underlying genes (Figure 1c) [45,46]. Their impact on AS regulation will be further discussed by us in other chapters. The RNA structure, yet still remaining elusive, have recently been given a greater consideration as new advances have partially overcome the difficulties associated with RNA structural dynamics, lifespan, and heterogeneity in eukaryotic cells, creating a whole-transcriptome landscape of RNA structures, the RNA structurome [47,48,49,50,51]. 

The aim of this review is to highlight recent findings exploring the phenomena of RNA structural arrangement, its cellular modulators and biological functionality linked to AS regulation and pathomechanism of splicing-associated diseases.

## 2. RNA Structural Arrangement

**Figure 2:**
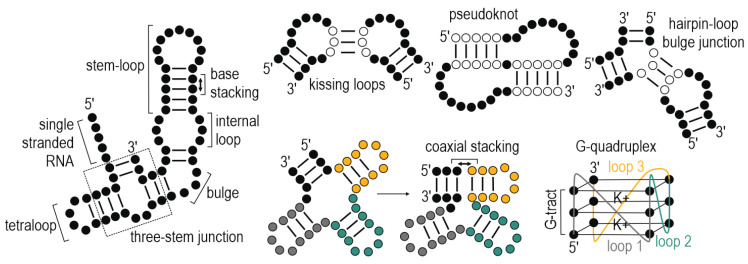
Schematic presentation of common RNA structural motifs present in secondary and tertiary structures. More details in the main text. Ribonucleotide residues are marked with black and white dots; hydrogen bonds are marked with short lines; base stacking is marked with a double arrow; K+, potassium ion.

The first discoveries of consensus motifs at exon-intron junctions were made in late 70′s [53]. Only a decade later the scientists proposed the link between the RNA secondary structure embedding these motifs and splicing regulation by implementing biochemical assays [54]. Thereafter, the biological function of RNA structure has been viewed by its propensity to form numerous preferential conformations enabling specific RNA-ligand or RNA-protein interactions. Rapidly developing and intensely studied field of RNA secondary and tertiary structures based on well-established biochemical, crystallographic, microscopic, and computational studies provided physicochemical principles of RNA folding which have been described in a number of excellent articles [55,56,57,58,59]. The composition of RNA linear sequence is a major factor driving the RNA folding owing to a high propensity of RNA bases and a backbone to interact with each other. It imposes the formation of either single-strands (ssRNA), more complex semi-stable secondary structures or double-stranded (dsRNA) regions attained through intermolecular forces such as hydrogen bonding underlying base-pairing and Van der Waals’ forces as well as hydrophobic effects responsible for stacking of adjacent bases (Figure 2) [55]. Nucleotides which organize into basic RNA secondary structural motifs including stem-loop structures, bulges, internal and hairpin loops and multi-stem junctions are further involved in intermolecular interactions underlying an arrangement of structural motifs of intricate shapes such as kissing loops, pseudoknots, hairpin-loop bulge junction, coaxial stacking of helices and RNA G-quadruplexes (rG4) [56,60] (Figure 2). The latter are highly stable structural motifs composed of a tract of stacking G-quartets through Hoogsteen hydrogen-bonding and sensitive to potassium ion (K+) concentration as well as molecular crowding [61,62]. RNA folding is substantially governed by pursuit of thermodynamic stability which in in vitro studies can be modulated by physicochemical conditions including ions’ concentration (K^+^, Na^+^, Mg^2+^), pH, and temperature [63,64], the factors fairly stable in eukaryotic cells [65]. 

Recent advances including high-throughput chemical footprinting combined with next-generation sequencing enabled to define remarkably detailed structural features of RNA in association with their biological role. Gracia and others provided a substantial body of evidence for incremental and concerted cooperativity between RNA structural motifs leading to folding of preferential RNA secondary and tertiary structures [66,67]. This cooperativity considers the formation of short-lived intermediates of RNA structure of less preferred thermodynamic stability which affect the kinetics of folding [67]. Xue and others utilized ^15^N relaxation dispersion nuclear magnetic resonance (NMR) combined with chemical probing to capture such intermediates of *p5abc* subdomain of the Tetrahymena group I intron ribozyme [68]. This substantial RNA feature impacts the efficiency of folding owing to the occurrence of additional pathways which may prevent the formation of nonnative, alternative RNA structures of diminished functions in living cells. It may also represent an adaptive response to the physiological and pathological conditions by safeguarding the genome from, e.g., deleterious mutations introducing alterations in RNA structural motifs. Another in vitro study performed by Lai and others using fluorescence resonance energy transfer (FRET) combined with computational analyses captured a final folding state of human mRNAs with short end-to-end distances supporting the notion of RNA high structure dynamics [69].

Considering given opportunities to form many energetically favored RNA structures exclusively to the native one, albeit at the expense of high energy- and time-consuming processes of RNA structural rearrangements, it is profound the way cells employ and manage factors introducing effective kinetics of functional RNA folding [70]. Therefore, there has been a continual necessity to confront collected knowledge from in vitro studies with a highly complex environment of living cells which provide a great range of additional factors like compartmentalization, concentration of biomolecules and protein-protein interaction network contributing to RNA conformation. 

The development of high-throughput RNA structure probing on a whole-transcriptome level brought an immense revelation in gaining a deeper insight into biologically relevant RNA structure information in vivo [71,72]. It was enabled through implementation of chemical reagents which rapidly penetrate into cellular compartments and selectively modify the exposed unpaired or flexible nucleotides. A few years ago, two studies provided the first comprehensive exploration of RNA structure in yeast and mammalian cells linking in vitro and in vivo RNA folding analysis [73,74]. Upon dimethyl sulfate (DMS) modifications, which interrogate adenosine and cytidine nucleotides, combined with new generation sequencing (DMS-seq), the authors found the mRNAs to be substantially less structured in rapidly dividing cells especially within coding regions than in vitro, most likely due to energy-dependent processes underlying RNA unfolding. Consistently, selective 2-hydroxyl acylation and profiling experiments (SHAPE) and DMS-based probing of transcriptome-wide rG4 structures in mouse embryonic stem cells (mESC) revealed reduced stability of these regions than in vitro studies [75]. As a complement of these findings, other research indicated that the occurrence of rG4 is most likely conditioned by sequence-context as proximal C-rich regions disfavored their formation [63]. Additionally, in silico consideration of existence of potential rG4 elements showed that they could have tremendous impact on both, the local RNA structural arrangement but more importantly on long-range interactions of distal RNA structural motifs. On the other hand, other examples of research showed a substantial level of human transcriptome structuration although differentiated between different classes of RNAs (e.g., protein-coding mRNAs, long non-coding RNAs, and enhancer RNAs). These RNA structural maps were obtained using in vivo click-selective SHAPE (icSHAPE) performed in mESC and with the use of low toxicity reagent called N_3_-kethoxal (albeit biased towards unpaired guanosine residues) [47,49,76]. In addition, transcriptome-wide identification of RNA duplexes in human cells illuminated a presence of long-range interactions and higher-order architecture across transcriptome [77,78,79]. 

Most research reflects the RNA structurome as the average RNA structures at steady state obtained in a whole-cell analysis that may limit our insight into the functional implications of RNA folding. This limitation has been partially overcome by achieving the mRNA structure dynamics during zebrafish development using DMS-seq analysis [80] or during cellular differentiation exploiting a psoralen crosslinking-based technique [78]. Another research exploited icSHAPE to differentiate the RNA structurome into three compartments: chromatin, nucleoplasm, and cytoplasm in human and mouse cells [50]. These results reflect RNAs to be slightly less folded in a nucleus and much more folded in intronic rather than exonic regions of pre-mRNAs compared to in vitro conditions. 

It needs to be considered that methodological aspects of experiments my underlie discrepancies between obtained results as some reagents show stronger bias towards sampling kinetically more stable or unfolded states of RNA structure or particular nucleotides during the time course of the experiment, whereas RNA structural arrangement undergoes transient and dynamic changes. Adding to the complexity, an important discovery was recently published showing much higher recruitment of RBPs to structured regions of transcripts [81], and thus probably limiting the access of structural probes to these regions. Additionally, utilized probes may diversely affect the physicochemical and biological modulators of RNA structure. Therefore, novel approaches may be essential to obtain comprehensive high-resolution information on RNA structurome in order to elucidate its biological role, with a particular attention given to low-abundance mRNAs excluded from high-throughput and whole-transcriptome studies. 

## 3. Cellular Modulators of RNA Structure 

The complexity of the cellular environment provides a great variety of dynamic modulators of RNA structure orchestrated by a spatiotemporal network of interactions. Their interplay results in a heterogeneity of the RNA structurome where RNAs are at different stages of the life cycle from transcription through translation and decay. Here, we will decipher selected modulators and their impact on RNA folding, whereas their involvement in mechanism of AS regulation will be extended in the next chapter.

### 3.1. Molecular Crowding

Cellular environment is characterized by molecular crowding underlain by a heterogeneous composition of inorganic components (e.g., cations, anions) and organic molecules (e.g., NTPs, RNA, proteins) of defined sizes and charge which entrain a steric hindrance due to their high accumulation and impenetrability [82,83]. This issue and its impact on macromolecules and biological processes have been covered in several excellent reviews [83,84,85]. What intrigues us, is that the molecular crowding vastly contributes to the stability of RNA structure and the kinetics of RNA folding by limiting RNA spatially [86,87]. In addition to observed accelerated folding and preferential stabilization of RNA structure, Dupuis and others discovered that the crowding-effect is driven by entropy changes [88]. The authors exploited a high molecular weight polyethylene glycols (PEG), a reagent commonly used to mimic the crowding-effect in vitro, and single-molecule FRET to explain the kinetics of conformational transitions for a GAAA tetraloop-receptor RNA [88]. The development of FRET based sensors relying on either a protein pair or oligonucleotides to examine the molecular crowding in eukaryotic cells emphasizes the significance of this phenomenon [89,90]. One particularly interesting finding reports lower crowding in a nucleus compared to the cytoplasm [90] that could partially contribute to the presence of less structured RNAs in this compartment reported by Sun and others [50]. Additionally, the nuclear crowding-effect appeared to be modulated by osmotic stress and drugs altering chromatin organization [90]. Thus, we could presume that any signals modifying molecular crowding in a nucleus may affect a broad spectrum of molecular processes including RNA folding and further RNA structure-dependent AS through, for example, modulation of the kinetic of reactions or local concentration and conformation of RBPs [91]. Apart from a rather disorganized crowding effect, the nucleoplasm compartmentalizes into particular biological process-oriented and phase-separated condensates with high density of distinct proteins and RNA substrates being continuously exchanged with adjacent environment [92]. For example, snRNPs are assembled and stored in so called Cajal bodies [93], whereas the transcription and to some extent splicing are constrained to nuclear speckles [94,95]. We can presume that these differential conditions within and outside condensates will have a distinct effect on RNA folding and consequently on functional relevance of RNA structural motifs.

### 3.2. Transcription

Cotranscriptional RNA folding has long been given a biological relevance [96,97,98]. A thorough description of mechanisms that have an impact on this phenomenon is comprised in exclusive reviews [98,99], whereas here we provide a brief introduction to this matter associated with AS and highlight the newest findings. Three crucial features are related to RNA folding during transcription: pausing of RNA polymerase, the elongation rate and cotranscriptional recruitment of RBPs to nascent RNA [97,98]. Considering that both the speed of eukaryotic RNA polymerase II (Pol II) and its propensity for pausing are gene- and locus-wise oriented, as well as coordinated by various RNA- and chromatin-driven mechanisms [100,101], the nascent RNA is given vastly differentiated time windows to achieve a functional folded state. With respect to cotranscriptional splicing [21], occurring either fast in yeast [102] or with diversified rates in metazoans [103,104,105], a nascent RNA is compelled to fold into a native and functional conformation available for and shape-shifted by partner molecules [97,98]. Contrarily, it could be assumed that different RNA folding states actually provide another regulatory layer in RNA processing as each folded structure either optimal, suboptimal, or entirely off-pathway structure contributes to the final downstream effect. Several in vivo studies, using slow and fast mutants of RNA II polymerase (Pol II) or drugs disturbing the Pol II speed, have indicated a profound effect of elongation rate on outcome of alternative RNA processing like splicing and polyadenylation [106,107,108]. This effect was mainly associated with features of the RNA linear sequence including the length of exons and flanking introns, the strength of splicing sites, the existence of auxiliary *cis*-acting elements and the kinetics of RBPs’ recruitment. Nevertheless, a growing body of evidence considers the significance of cotranscriptional RNA folding in RNA processing. Due to such limitations as the speed of Pol II (~1–4.6 kb min^−1^) [109] and the time required for experimental nascent RNA probing, the direct monitoring of dynamic cotranscriptional RNA folding is vastly challenging. The first transcription-wide scale analyses of cotranscriptional RNA structure formation was achieved in prokaryotic cells by introducing a new method called structural probing of elongating transcripts (SPET-seq) relying on parsing the transcription intermediates [110]. The authors have shown immediate and transient formation of short-range interactions of newly transcribed RNAs and the occurrence of intermediate RNA structures for long-range interactions consistently with aforementioned in vitro captured intermediates [67,68] and the propensity to form structural motifs by RNA [56]. Contrarily to prokaryotes, the area of research related to cotranscriptional RNA folding in eukaryotes is much less explored. One of recent findings was achieved by Saldi and others, who performed chemical probing of nascent RNAs [111]. They observed disturbance in RNA secondary structure folding upon activity of slow Pol II leading to a failure of proximal histone mRNA 3′ end processing. In fact, in terminally differentiated cells a subset of long polyadenylated mRNAs occurs naturally what could suggest some kinetic changes of transcription machinery linked to development or even aging [112]. On the other hand, another study conducted in human and mouse cells revealed the RNA structurome to be more structured upon lower transcriptional rate as it most likely lengthens the window time for RNA folding and local accumulation of RBPs [50]. An important insight into the dynamic biomolecular reaction networks involving RNA metabolites, RNAs and proteins during in vitro transcription was provided by Nikolaev and others [113]. The newly invented method called Systems NMR enables to track each component of the system and study various reactions concurrently over time and at different conditions. One of the analyzed biological processes was interaction between hnRNP A1 and two short structured RNA molecules in two configurations, in the course of the transcription and post-transcriptionally. The protein was found to bind and unwind the RNA hairpin of the first RNA sequence during transcription, but also to form complexes with a stem-loop of the second RNA which were stoichiometrically distinct in those two configurations. Thus, RNA folding may affect AS regulation by for instance determining the nature of RNA-protein interactions. All these studies indicate that the cotranscriptional RNA folding constitutes a dynamic and intricate process with a biological relevance yet to be more characterized in eukaryotes. 

### 3.3. RNA Modifications, Editing and Sequence Composition

Among dozens of internal RNA modifications shape-shifting RNA structural arrangement, methylation of adenosines and isomerization of uridines to pseudouridines are the most ubiquitous in a nucleus, albeit still perplexing researchers with their role in gene expression regulation. The *N*^6^-methylation of adenosines (m^6^A) is deposited at RRA*CH (R, purine; A*, methylatable A; H, non-guanine base) consensus sites by the activity of methyltransferases like 3 and 4 (METTL3 and METTL4, respectively) [114,115]. Both in vitro and in vivo studies arose m^6^A-imposed RNA structural regularities associated with destabilization of RNA duplexes [47,116,117] and potentially stabilization of ssRNA regions through stacking [117]. In consequence, m^6^A-induced location-dependent switch of RNA secondary structure may recruit m^6^A ‘reader’ proteins or increase the accessibility of the adjacent RNA motifs for splicing factors or, in contrast, m^6^A may be recognized and removed by ‘eraser’ proteins; all these mechanisms are substantial in coordination of several aspects of RNA metabolism predominantly AS [118,119,120,121,122,123,124,125]. The m^6^A deposition is most likely driven before or soon after the occurrence of exon definition in nascent pre-mRNA consistently with recent findings showing a strong correlation between AS regulation and enrichment of m^6^A signals within introns or their reduction at splice junction exonic boundaries [126]. The mechanism of AS regulation through m^6^A-driven RNA structural arrangement is further extended in the next chapter. 

Pseudouridines (Ψ) are widely abundant modifications in eukaryotes added primarily by the activity of standalone pseudouridine synthases (PUS) and Box H/ACA ribonucleoprotein enzymes in the course of transcription [127,128]. Not until recently, have the principles of RNA target recognition by PUS been revealed emphasizing RNA-structure driven activity of this synthetase in vitro and suggesting the requirement of particular RNA folding prior to pseudouridylation in vivo [129]. The authors developed an in vitro, high-throughput pseudouridylation assay in yeast, illuminating the significance of HRU (R, purine; H, non-guanine base) sequence motif to be embedded in a bulged stem-loop structure to serve as a target of PUS1. Interestingly, the biochemical assays showed that Ψ serves as a structure-remodeling and versatile base owing to its propensity to stabilize the conformation of RNAs and interact with four ribonucleotide residues [130,131]. Nevertheless, the in vivo RNA structurome analyses revealed less structured Ψ-containing regions rather than predicted in in vitro assays [50] emphasizing the complexity of cellular environment and presence of a wide range of diverse RNA-structure altering factors. Ψ moieties are enriched in noncoding RNAs [132], for example, in major spliceosomal snRNAs where they play an important role in splicing regulation at the level of proper RNA-RNA structure formation and RNA-protein interactions [133]. Our knowledge on the extent of pseudouridylation of protein coding RNAs has increased in the last decade but there are still many open questions concerning their mechanism and biological function [134]. Direct readers of Ψ or Ψ-imposed RNA conformations or erasers are essential to be revealed to elaborate the functional importance of Ψ in gene expression. 

Adenosine-to-inosine (A-to-I) RNA editing conducted by adenosine deaminase acting on RNA (ADAR) is another RNA shape-shifting factor whose activity leads to unwinding of RNA duplexes as A-U Watson-Crick base pairs are converted to I-U wobble pairs [135,136]. Its initially discovered role was assigned to translation regulation as inosine is interpreted as a guanosine by the ribosome, whereas the editing itself was believed to occur mostly post-transcriptionally [136]. Further extensive analyses confirmed cotranscriptional editing and tightly coupled its functional importance with splicing by describing the interplay between ADAR itself, ADAR-driven disturbance of RNA structure or a sequence motif of *cis*-acting sites and splicing machinery or auxiliary factors [137,138,139,140]. Given that endogenous editing efficiency is transcript-specific and occurs in a variable manner for each transcript copy, it suggests a complex and multilayered regulation of ADAR activity. In fact, Daniel and others discovered the presence of a supporting dsRNA editing inducer element adjacent to the actual target site which most likely recruits ADAR and increases its local concentration enabling the reactions [141]. In contrary, DExH-Box Helicase 9 (DHX9) may directly counteract or promote ADAR’s activity through structural remodeling of its RNA substrate [139]. 

Due to the fact that the fidelity of adenosine methylation, pseudouridylation, and RNA editing depends on linear and/or structural RNA motifs and that they are engaged in gene expression, the dynamics of RNA modification stoichiometry and their kinetic timing during RNA maturation comprise another crucial layer of regulation which remains to be deeper characterized.

Intriguingly, ADAR activity is tightly associated with primate-specific *Alu* short transposed elements which may be distinguished from a linear sequence and perceived as specific *cis*-acting modulators of RNA structure. *Alu*s appear in tandem inverse orientation and tend to form intermolecular dsRNA structures subjected to ADAR editing [142,143]. They are prevalent in introns within gene-rich regions of the human genome [144], especially upstream to alternative exons where their pairing affects AS, however the mechanism behind this remains obscure [145,146,147]. They also provide splice acceptor sites [148], which may be alternatively selected upon RNA editing followed by RNA structure alterations and recruitment of splicing machinery [144,145]. Analogously to *Alu* elements, short complementary runs of nucleotide repeats are reported to play an essential regulatory role [42]. Lin and others deciphered AC and GT-rich tracts which mediate a highly stable RNA structure across a particular class of introns enforcing splice site selection and resulting in splicing determination in fish and lamprey [42]. The authors take a step forward identifying such elements in mammals suggesting the functional role of G-, C-, and GC-rich repeats favored within thermodynamically more stable introns. Such a link between nucleotide composition bias around splice sites and AS outcome has been determined by extensive studies; for instance high GC content may promote the formation of stable secondary structures and therefore either reduce exon recognition or enhance it by recruiting splicing factors to GC-rich motifs preceded by the activity of RNA shape-shifting protein factors [40,42,149,150,151]. 

### 3.4. RNA Structural Switches

Alterations in RNA structure in response to environmental and cellular signals constitute one of the signaling pathways and executors of gene expression regulation. These signals in a form of particular metabolites are recognized by aforementioned riboswitches [45,46]. They are extensively studied in prokaryotic cells due to their high abundance and outstanding contribution to directing transcription and translation in various metabolic, physiological, and pathological pathways [45]. The only known class of eukaryotic riboswitches found in fungi, archaea and plants resides within intronic regions of TPP metabolism genes and fine-tunes their AS and expression in response to TPP binding (Figure 1c) [52,152,153,154,155,156]. 

In case of the majority of eukaryotes the protein- and nucleic acid-directed RNA structural switches predominate. They enable stabilization or formation of functional RNA secondary structures through transient or stable interactions and, in turn, lead to a functional response. Their thorough characterization has been captured in several reviews [51,157,158,159]. Here, we will focus on their specificity and function in relation to AS. 

Helicases comprise the largest group of transient remodelers of DNA and RNA structural arrangement, categorized into superfamilies and families and involved in virtually every aspect of DNA and RNA metabolism at the expense of ATP [160]. The activity of RNA helicases, especially two main DEAD-box (DDX) and DExD/H-box families, may substantially differ. Some of them translocate along an RNA strand, unwind RNA duplexes, and displace proteins, while others are capable of solely unwinding dsRNA regions and/or mediating RNA-annealing [160,161,162]. Although, in general, they are expected to bind a dsRNA target in a sequence-independent manner, recent findings indicate distinct RNA sequence and structure preferences enabling their loading and ATP-hydrolysis including rG4, GC-, C- and CU/CA-rich motifs [163,164,165,166,167,168], or even a requirement of an auxiliary ssRNA region adjacent to a putative dsRNA target [166]. Regarding pre-mRNA splicing, RNA helicases may couple with spliceosome components, large assembly of splicing regulators (LASR) or auxiliary *cis*-acting elements altering RNA-RNA interactions and remodeling RNA-protein complexes [166,167,169,170,171].

Another largely heterogeneous class of RNA structureswitches, RBPs, binds and forms a stable but not always functional complexes with RNA in an energetically independent manner. Upon binding, the RBPs exert local RNA structural alterations as well as changes of structural context of adjacent regions due to torsional stress and thermodynamic compensation of local alterations. A recent whole-transcriptome analyses of RNA structurome enabled to decipher this issue in a broader context. The authors noticed that the occupancy of many RBPs is linked to RNA structural arrangement either in favor of stabilization or destabilization of RNA structures. For instance, the chromatin-associated proteins tend to interact with less structured RNA regions which undergo folding once they dissociate from the chromatin and the ribonucleoprotein (RNP) complexes [50]. A double-stranded RNA-binding protein Staufen homolog 1 (STAU1) was found to stabilize RNA structures upon binding after RNA leaves chromatin, whereas hnRNP C binding to RNA was correlated with the structural disturbance of flanking regions [50]. The aforementioned AS factor, MBNL1, forms functional complexes with specific linear and structural RNA motifs [172] but upon binding it most likely unwinds the local secondary structure of the RNA as it was shown in an in vitro footprinting assay [173]. 

The class of RNA-mediated RNA structure switches engaged in RNA processing is rather scant, but it is worth mentioning C/D box small nucleolar RNAs (SNORDs) which are 60- to 300 nt-long non-coding RNA species derived from excited introns and accumulated in a nucleolus [174]. One of their subsidiary and newly described roles is regulation of AS. Several pre-mRNAs were confirmed to be SNORDs substrates and dozens of them were selected as potential targets [174,175,176]. Mechanism of their function relies on a stretch of complementary pairing within 5′ss regions which become double-stranded and unavailable for splicing components. 

## 4. Mechanism of Alternative Splicing Regulation by RNA Structural Conformation 

**Figure 3:**
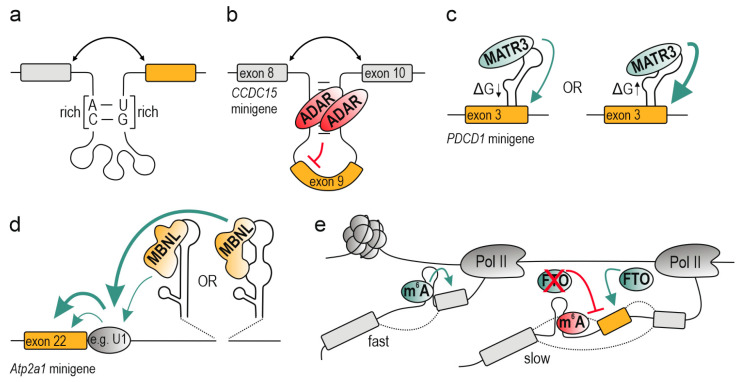
Schematic diagrams showing different mechanisms of AS regulation by RNA structural arrangement. RNA structural arrangement may mediate AS via: (**a**) bridging *cis*-acting elements [42]; (**b**) looping out alternative exons or *cis*-acting elements [140]; (**c**) blocking/promoting interaction with splicing factors [167]; (**d**) allosteric activation/inhibition of splicing factors [172]; (**e**) modulating the splicing kinetics [126]. Detailed explanation can be found in the main text. Alternative exons are in orange; constitutive exons are in grey. *CCDC15*, coiled-coil domain containing 15 transcript; MATR3, Matrin-3 protein; ΔG, the change in Gibbs free energy, serves as a measure of thermodynamic stability of RNA secondary structure; *PDCD1*, programmed cell death 1 transcript; *Atp2a1*, ATPase sarcoplasmic/endoplasmic reticulum Ca2+ transporting 1 transcript; U1, small nuclear ribonucleoprotein U1 (U1 snRNP); Pol II, RNA polymerase II; FTO, RNA m^6^A demethylase.

Up to that point, we addressed particular *cis*- and *trans*-acting factors underlying dynamic and intrinsic alterations of RNA structural arrangement in a cellular environment. The same factors may utilize distinct mechanisms to coordinate AS, which overlap or complement one another with a vague borderline between them. Below, we discuss the main RNA structure-mediated mechanisms of AS including bridging or looping out *cis*-acting elements, blocking or promoting interaction with splicing factors or their allosteric activation/inhibition, modulating the splicing kinetics (Figure 3).

### 4.1. Bridging Cis-Acting Elements 

Spatial closeness of spliceosome components plays a substantial role in orchestrating efficient splicing. A strong evidence is exemplified by recursive splicing in which excessively long introns are exclusively processed in a stepwise manner owing to the presence of non-canonical splicing sites located deep within introns [177]. As early as in 1997, Howe and Ares identified intronic sequences of high complementarity in yeast, proposing a model in which their pairing brings closer 5′ss and branch point and enables inclusion of a downstream exon [178]. Consistently, other studies in yeast describe the formation of a stable stem-loop structure bringing into closer proximity the *cis*-acting elements which was found essential for both constitutive splicing [179,180] and AS in response to heat shock [181]. Grasping this phenomena from a wider perspective, from the high-throughput chemical probing of the RNA structure and computational analysis emerged a higher-order structural organization of the RNA structurome with long-range alternative RNA-RNA interactions in mouse and human cells, which could serve as putative bridges for alternative exons that are separated by hundreds or thousands of nucleotides [77,78]. Undertaking computational and enzymatic approaches AC- and GU-rich RNA structures were discovered on the boundaries of a subset of introns in fish [42] (Figure 3a). By employing several splicing minigenes and mutagenesis the authors confirmed their highly significant effect in splicing regulation most likely due to bringing together the splice sites. Genome-wide crosslinking, immunoprecipitation and deep sequencing studies performed in mouse and human cells indicated a supportive intronic stem-loop structure which bridge a distal binding site for RBFOX with an alternatively regulated exon [182]. The formation of paired intronic complementary sequences has also been a crucial determinant for alternative backsplicing, since their formation mediates the selection of proximal and distal 5′/3′ back-splice sites [9]. Riboswitches and *Alu* elements, due to their natural propensity to form long-range RNA–RNA interactions, may also play a significant role in distance dependent splice sites recognition [145,153].

### 4.2. Looping out Splice Sites and Entire Exons

Apart from bridging splice sites and other decisive regulatory elements, the RNA duplex-formation may lead to their looping out which subsequently excludes these elements from a splicing process. One of the first observations displayed the hnRNP A1-induced RNA structure formation which triggered looping out of internal 5′ss; however, an equivalent result was obtained in a protein-independent approach by inserting RNA duplex-forming inverted nucleotide repeats into the minigene replacing the natural hnRNP A1 binding site [183]. Another described RNA remodeler, PTBP1, silences exon inclusion by bringing in close proximity two polypyrimidine tracts leading to looping out *cis*-acting elements or entire alternative exons [184]. Miriami and others conducted computational analyses which led them to identify dozens of alternatively skipped exons to be flanked by GC-rich sequences forming stem structures in human cells. In consequence, these exons were expected to be looped out while upstream 5′ss and downstream 3′ss were brought together and preferentially selected [149]. Accordingly, RNA long-distance interactions and subsequent looping out of alternative exons may be mediated by ADAR, independently on its editing activity, as it was proposed for regulation of alternative exon 9 of pre-mRNA of coiled-coil domain containing 15 (*CCDC15*) [140] (Figure 3b). Coordination of AS by long-range interactions was also confirmed in multiple *Drosophila melanogaster* pre-mRNAs [185]. This regulatory scheme is discerned in splicing mechanism of mutually exclusive exons (MXE). The main principle of splicing of MXE relies on competition between multiple and complementary structural elements which serve as selector sequences and docking sites [186,187]. They are mainly positioned within introns and bridge together distinct *cis*-acting elements presenting only one exon from the cluster of MXEs to the spliceosome at a time while others are looped out. A leading example comprises *Drosophila melanogaster* Down syndrome cell adhesion molecule (*Dscam1*) transcript carrying four exon clusters, including multiple MXEs which give rise to nearly 40,000 isoforms [185,188,189]. 

### 4.3. Blocking (Steric Hindrance)/Promoting Interaction with Splicing Factors

As early as in 90′s the propensity of splicing factors to regulate individual alternative events was linked to the RNA structural context of *cis*-acting elements [190,191]. Since then, numerous and single gene-oriented studies evoked the regulatory potential of RNA structure to inhibit or promote the interaction of splicing factors with pre-mRNA [172,192,193,194]. In fact, the summary studies of dozens of crystallographic RNA-protein complexes highlighted the importance of structural arrangement of protein and RNAs at interaction surfaces over sequence-specificity with a bias towards availability of ribonucleotide sequences within single-stranded conformation in sequence-specific and nonspecific interactions [195,196,197]. Consistent with the fact that most RBPs interact with ssRNAs, a thorough analyses of published *cis*-acting splicing enhancers and silencers showed a strong correlation between their single-stranded arrangement and splicing regulatory activity [39].

The development of whole-transcriptome methods and high-throughput in vitro and in vivo approaches in the last decade provided an intrinsic transcriptome-wide RNA-protein interaction map. These methods include predominantly in vivo crosslinking and immunoprecipitation combined with deep sequencing (CLIP-seq), its derivatives or a RNA Bind-and-Seq (RBNS) assay [198,199,200]. As expected, the majority of protein-recognized RNA cognate motifs within the transcriptome turned out not to be occupied by RBPs [201,202] providing RNA structure context as the key binding determinant [32,197,198,201,203]. In support of this notion, structured regions within all RNA species were recently found vastly more favored for RNA-protein interactions and the level of structural arrangement correlated with the amount of bound proteins and the significance of a transcript in control of cellular networks [81]. Notably, different types of methods mapping RBP-binding sites along with gene expression data are nowadays substantially exploited by in silico approaches and serve as a platform for modeling and predicting binding preferences of RBPs combining RNA linear motifs and their structural properties (e.g., GraphProt, RNAMotifs, SMARTIV, RNAcompeteS) [204,205,206,207]. In Table 1, we combined information on selected alternative splicing factors and their binding preferences towards RNA linear and structural arrangement. 

An interesting example of RNA structure-mediated interaction between splicing factors was described by Warf and others [232]. The authors noted a competition between MBNL1 and U2 small nuclear auxiliary factor 65 kDa (U2AF65) for splicing control of cardiac troponin T (*cTNT*) exon 5 mediated by two mutually exclusive RNA structures. The binding region for these splicing factors folds into either a single strand or a stem-loop structure enabling only one of these proteins to bind. Correspondingly, Sun and others have recently described RNA structure-mediated control of AS of programmed cell death 1 (*PDCD1*) alternative exon 3 [167]. In this research, a series of biochemical and functional studies have shown an exonic GC-rich stem-loop structure adjacent to 5′ss of intron 3 to recruit a positive splicing factor Matrin-3 (MATR3) which bound UCAUCU auxiliary motif within the loop and promoted exon 3 inclusion (Figure 3c). This splicing effect was deepened in consequence of structural destabilization of the stem-loop via mutagenesis (an increase of ΔG value). Contrarily, the stem element of the structure was also shown to recruit DDX5, which exerted a negative effect on splicing; however, the exact mechanism of DDX5 regulation requires deeper exploration.

AS control by G-rich elements residing nearby alternative events has been well covered in research [233,234,235,236]. However, the collected knowledge of direct readers of G-containing assemblies still remains scarce. Some biochemical and cellular assays brought divergent evidence supporting hnRNP F/H recognition of either a stable rG4 [237,238,239,240] or solely a single-stranded fraction of G-rich tracts perhaps cotranscriptionally prior to folding [241,242]. One of explanations could be drawn that in vitro conditions do not reflect the complexity of cellular environment in which multiple known and yet to be revealed factors may affect such features of tested components as the native structure and biological activity, heterogeneity, the kinetics of complex formation and cooperativity with other factors. This is demonstrated for RNA helicases which mediate rG4s unwinding and enable other factors to run various processes [243]. Considering splicing, Dardenne and others exposed an intriguing cooperation between hnRNP F/H and RNA helicases in G-rich tracts recognition and AS regulation during muscle differentiation and the epithelial-mesenchymal transition (EMT) [150]. These results allowed to surmise that DDX5 and DDX17 facilitate hnRNPs interaction with these otherwise structured G-rich motifs through their unwinding. A corresponding coordinated and guanine-mediated regulation of common alternative events was noted between DDX5 and several other splicing factors including RNA binding protein 4 (RBM4) [244], MBNL1 [245], hnRNP A1 [166], MATR3 [167] as well as between another helicase DHX9 and ADAR [139]. The latter research indicates binding sites for both factors to be enriched in GC-rich elements expected to form duplexes and with adjacent ssRNA regions perhaps acting as a loading platform for DDX5. A direct interaction of certain hnRNPs, e.g., hnRNP U, with Pol II and chromatin remodelers could also suggest higher local concentration of splicing factors at nascent RNAs increasing a chance of their binding to yet unfolded state of RNA [246]. Other examples of the rG4-related AS control pathway were described for alternative exons of several transcripts including pre-mRNA of B-tropomyosin [247], Paired box gene 9 (*PAX9*) [248], p53 [234] or fragile X mental retardation 1 (*FMR1*) [249]. In the latter, the exonic rG4s act as ESE in a negative autoregulatory loop through recruitment of FMRP protein which may subsequently modulate the function of putative splicing factors. 

The m^6^A modification may orchestrate the access to regulatory elements through the impact of the modification itself on RNA-substrate recognition by RBPs [126,250] or through m^6^A-imposed RNA structure switch affecting the recognition of RNA regulatory elements by RBPs [50]. The latter was underlain by high-throughput analysis of structural context of ADAR-edited regions which co-occurred with alternative events [251]. The structure switch of RNA regulatory elements was found decisive for the AS coordination by several splicing factors such as hnRNP C [50], hnRNP G [120], and hnRNP A2/B1 [252]. Recent findings report that binding of ADAR itself to dsRNA regions formed between GA-rich sequences and Py-tract governs AS through either sterically precluding access of U2AF65 to nearby Py-tract or by masking the splice sites [137,140]. 

*Alu* elements could also act accordingly. Indeed, there are several lines of experimental evidence that ADAR-associated *Alu* elements, due to their propensity to form long-distance structures, are also capable of occluding the splice sites or preventing their recognition by splicing machinery [143]. Consistently, several studies undertaking computational and cellular approaches identified that enrichment of intronic *Alus* comprises a substantial determinant of splicing profile of adjacent alternative exons which could suggest a great importance of long-distance interactions within RNA in regulation of AS [145,146,147]. An illustrative example of a vastly complex and cooperative RNA structural arrangement which governs AS is present within survival motor neuron 2 (*SMN2*) transcript which is critical for development of spinal muscular atrophy (SMA), a genetic disease fatal at early age [253]. A unique protein-independent long-distance interaction within intron 7 has been discovered and confirmed in chemical structure probing to form between the first several nucleotides of intron 7 and a region downstream, distant by nearly 300 nt [254,255,256]. This deep intronic sequence occludes 5′ss and nearby ISE leading to exon 7 exclusion by inhibition of recruitment of U1 snRNP and a positive splicing factor, T-cell intracellular antigen 1 (TIA1) [255,257]. Among other structural motifs, the one positioned at 5′ end of exon 7 forms a stem-loop structure with additional inhibitory effect on U1 snRNP recruitment [258]. These and other findings culminated in designing a therapy for SMA further described in the next chapter [259].

Regardless of the extensive pseudouridylation of snRNAs and its role in spliceosome assembly, little is known about the mechanism of splicing through pseudouridylation of pre-mRNA [133,134]. Potentially Ψ could contribute to splicing outcome through imposed RNA structure stabilization within decisive pre-mRNA regions disabling their recognition by splicing factors. Extensive studies are essential to support this notion.

In fungi and plants, the RNA structural rearrangement of splicing sites modulating their availability for the spliceosome was found to occur in the context of TPP-sensing riboswitches [52,152,154]. For instance, a fungal riboswitch located within a *nmt1* pre-mRNA comprises an intronic TPP binding cassette which, according to in-line probing experiments, partially base pairs with and occludes an alternative 5′ss, acquiring the “ON” state and allows for translation of nmt1 protein [52] (Figure 1c). In consequence of TPP binding, the large structural rearrangement is believed to enable the selection of alternative 5′ss by the spliceosome with concomitant repression of a branch site, resulting in reduction of *nmt1* expression. Intriguingly, Gong and others provided even deeper elucidation of the kinetics of TPP-riboswitch structural response by exploiting a systematic helix-based computational method [260]. The authors proposed a co-transcriptional mode of TPP binding before the riboswitch folds into thermodynamically optimal “ON” state in order to facilitate the otherwise extremely slow and energetically excessive structure transition.

### 4.4. Allosteric Activation (Enhancement)/Inhibition (Deterioration) of Splicing Factors

The diversity of RNA structures and their coexistence driven by multiple cellular factors, constitute a regulatory layer of RNA-protein interactions similarly to optimal and suboptimal linear motifs with varying degrees of their effect on AS [198,201,203]. On one hand, the formation of RNA-protein complexes may require clearly defined structural arrangement of RNA and/or proteins [261,262]. On the other hand, the RNA-protein interaction may be dictated by wide-ranging conformational criteria. Accordingly, the RNA substrate may impose distinct structural changes in proteins upon their binding [263]. Whether RNA structure enabling docking differs from RNA structure enabling particular protein activity remains still an intriguing hypothesis to be tested. Disordered regions of RBPs (enriched in arginine, glycine, serine, and lysine residues) are an example of the most prone to conformational changes; in consequence, upon RNA binding or posttranslational modifications they may undergo disorder-to-structure transition affecting the activity of the protein [184,264,265,266]. Shedding more light into structure-driven protein interactivity, recent findings have shown the preference of disordered and polar proteins to bind ssRNA, whereas structured and hydrophobic proteins favor dsRNA [81]. Besides their prevalent function in mediating RNA binding, disordered regions are often involved, cooperatively or competitively, in protein–protein interactions [265]. For instance, highly disordered linker sequence of MBNL1 which orientates two tandems of RNA binding domains with respect each other enables both efficient interaction of the protein with RNA as well as activation and repression of alternative events [267,268]. Whether the linker serves as a platform for protein–protein interactions still remains to be experimentally investigated. Nevertheless, we recently studied the effect of RNA structure embedding a few 5′-YGCY-3′ motifs on MBNL1-RNA complex formation and its downstream functional impact on MBNL-dependent splicing by exploiting biochemical assays and a subset of splicing minigenes [172] (Figure 3d). We noted that subtle mutation-induced changes in structural arrangement and location of MBNL-recognized sequence motifs had a plethora of distinct effects on splicing efficiency of the alternative exon but not MBNL1 affinity. We surmise that MBNL1 binding to distinct RNA structures could mediate conformational changes of disordered regions, which either serve as splicing domains or affect those and thus modulate the MBNL1 splicing activity due to altering the protein–protein interaction surface. However, we cannot rule out the possibility that distinct suboptimal RNA structures may also alter binding kinetics or RNA-protein complex stability. 

### 4.5. Modulating the Splicing Kinetics

Even though there is no strong or direct evidence, the model of RNA structure interference into splicing kinetics has been proposed to mediate AS outcomes. 

In yeast, the cotranscriptional RNA folding was predicted to substantially influence transcriptional elongation rate, which, in turn, determines inclusion or skipping of alternative events [104,269,270]. Based on a single-molecule in vitro transcription assay and cotranscriptional folding simulation it was rational to surmise that stable RNA structures, mainly GC-rich, promote transcription elongation by structure-dependent impeding of RNA Pol II backtracking (proofreading step) and pausing along the template. Reversely, nascent RNA of lower structural stability close to the polymerase was associated with slowed and paused Pol II. The newest findings, supporting these observations, extended our comprehension of a regulatory potential of RNA structure in splicing modulation through affecting Poll II elongation rate [270]. As modeled by the authors, cryptic and alternative 3′ss are more prone to be preceded by stable RNA structures and omitted by spliceosome due to most likely RNA-structure mediated hastening of Pol II. We could also conjecture that the rate of cotranscriptional RNA-folding may influence recruitment of splicing factors and their local concentration, and in this way coordinate the splicing kinetics. 

It has been suggested that *Alu*-mediated structure disturbance occurring close to splice sites may further diminish their recognition by splicing components and affect kinetics of their binding leading to slower splicing kinetics or suboptimal exon selection [145]. Likewise, dsRNA driven m^6^A modification has been recently correlated with splicing kinetics of alternative exons either through direct recognition of m^6^A by readers or indirectly through m^6^A-imposed RNA structural rearrangements [126]. The authors observed that deposition of m^6^A by METTL enzymes at exon-intron splice junctions was strongly linked to fast splicing kinetics and constitutive splicing (Figure 3e). On the other hand, the intronic enrichment of m^6^A was vastly correlated with slow-processivity of these introns and occurrence of alternative exons. From further studies emerged an interplay between intronic deposition of m^6^A and RNA m^6^A demethylase, FTO, in AS control. m^6^A removal by FTO was associated with alternative exon inclusion, whereas FTO depletion led to increased exon skipping.

## 5. Splicing-Related Diseases Mediated by RNA Structural Arrangement

RNA structural arrangement and AS have been extensively studied leading to an increasing understanding of the role of their functional crosstalk in pathogenesis and progression of various diseases as well as development of potential therapeutic strategies [271]. Apart from a direct influence of certain hereditary or somatic mutations, also called riboSNitches, on RNA structural conformation, the disturbance of RNA structure caused by RBPs and impaired interplay between them constitute a great and vastly heterogeneous regulatory layer which contributes to RNA structure-engaging disease development. Here, we will decipher and discuss the role of AS mediated by RNA structural arrangement in pathogenesis and progression of selected groups of diseases, including illustrative examples. 

### 5.1. Diseases Associated with Single Nucleotide Variants (SNV)

The development of large-scale sequencing approaches and big data analytic tools with a single nucleotide precision opened a wide window for diagnostics of genetic disorders and identification of putative deleterious mutations through screening of individual human genome and exome [272]. In comparison to the advancement of these tools, equally important studies leading to identification and functional characterization of causative mutations are rather underrepresented. In some cases, the effect of mutations on a molecular mechanism of a disease may be quickly determined especially if a mutation disrupts a splice site or introduces a PTC which manifests in Duchenne muscular dystrophy (DMD) or cystic fibrosis (CF), respectively [273]. Contrarily, the role of mutations residing in stretches of noncoding regions or outside of known regulatory elements is more challenging to explore, especially if mutations affect the structural arrangement of local or long-distance RNA interactions. Different software may be applied to overcome some obstacles by, for example, predicting the pathogenic effect of SNVs on AS in human genome [274,275,276]. A computational analysis of disease-associated SNVs within UTRs revealed SNV-mediated local and global alterations of structural arrangement of these regions most likely significant in pathomechanism of, for example, β-thalassemia and chronic obstructive pulmonary disease (COPD) [277]. Giving consideration to splicing, it is noted that about 30% of disease-associated mutations disrupt splicing, whereas 25% of these mutations occurring within exons mediate exon skipping either through disturbance of ESEs in, e.g., Stickler syndrome or enhancement or creation of alternative ESSs in, e.g., SMA [273,278]. Intriguingly, the intronic disease SNVs, which mediate deep splicing changes, often reside far from splice sites potentially affecting the sequence motif of ISE or ISS or introducing disfavored structural changes [279]. Recent studies have utilized a novel approach called massively parallel splicing assay (MaPSy) to characterize a sequence context and mechanism of SNVs through screening of nearly five thousand of disease-associated exonic mutations derived from the Human Gene Mutation Database (HGMD) [43]. The effect of mutations on splicing was evaluated in both in vivo and in vitro approaches showing 10% of overlapping events and in reference to their impact on assembly of subsequent spliceosome complexes. Interestingly, a selected group of RNA samples with mutations stabilizing the RNA secondary structure inhibited each step of spliceosome assembly, compared to other RNA sequences with mutations disrupting *cis*-acting sites which stalled in early or later spliceosome complex. Thus, this effect on splicing is thought to be RNA structure-mediated and independent of *trans*-acting factors as well as tissue and cell-type nonspecific. 

On the other hand, the SNV-based structural alterations of regulatory elements may also change their accessibility for *trans*-acting factors and functionality. One of the widely examined and heterogeneous groups of SNV-associated neurodegenerative diseases, tauopathies, comprises well known Alzheimer’s disease (AD) and a class of frontotemporal lobar degeneration (FTLD) [280]. They are underlain by hyperphosphorylation, pathological misfolding and aggregation of tau proteins inside neurons of yet an unclear underlying mechanism [281]. However, the disease SNVs within alternative exons 2, 3, and 10 of microtubule-associated protein tau (*MAPT*) transcript, which causes *MAPT* pre-mRNA missplicing, were found in patients and might contribute to the pathomechanism [281]. Particularly, exon 10 encodes one of four microtubule binding domains (MBD) which bind to and stabilize microtubules [282]. Disruption of ratio between tau proteins with three and four MBDs in neurons is generally related to higher affinity of tau to microtubules and manifests in frontotemporal dementia with parkinsonism-17 (FTDP-17) [280,283]. Various mutations in the vicinity of 5′ss of exon 10 increase U1 snRNP binding leading to enhanced production of four-MBDs containing tau proteins [280,284]. Successive discoveries indicated a natural regulatory RNA stem–loop structure embedding 5′ss which due to disease SNVs undergo U1 snRNP-favored destabilization [283,284,285]. 

An interesting example of a disease in which SNVs and RNA structural arrangement play a critical role in therapeutic strategy development is SMA, a recessive genetic neurological disease. In general, the pathomechanism of SMA is underlain by disease-associated deficiency of SMN1 protein, while the production level of its paralog, SMN2, is naturally low and insufficient to compensate for SMN1 [253,286]. In consequence of SMNs’ cellular insufficiency several processes are disrupted including pre-mRNA splicing, what further leads to degeneration of motor neurons in the spinal cord and muscular atrophy. *SMN1* and *SMN2* genes differ from each other by the presence of deletions and substitutions leading to *SMN2* exon 7 exclusion and expression of a truncated and only partially functional SMN2 protein due to in-frame occurrence of PTC within exon 8 [253,287]. Several initial studies discovered a few splicing factors both enhancers and silencers implicated in exon 7 regulation and being affected by a particular SNV, the C-to-T substitution, at 6th nucleotide (C6U in RNA) of exon 7 [253,288]. In addition, this SNV was found to stabilize a stem-loop structure present in the vicinity of 3′ss of exon 7, adding another regulatory layer to *SMN2* splicing regulation [289]. Until now, the regulation of exon 7 has been linked with cooperative and inhibitory interplay between nearly 40 splicing factors and a vastly sophisticated structural arrangement of exon and intron 7 involving long-distance interactions [253]. A great amount of often arduous work, which profoundly increased our knowledge on the mechanism of *SMN2* exon 7 splicing, culminated in the design of antisense oligonucleotide-based therapeutics. Spinraza^TM^ is a drug approved in 2016 by Medical Drug Association (MDA), which is complementary to a *cis*-acting element within intron 7 called Intronic Splicing Silencer N1 (ISS-N1), and abrogates its negative effect [290,291,292]. Another potential drug has been thus far tested in vivo and shown to ameliorate symptoms of SMA in mice by a moderate increase of SMN2 production as a result of targeting a 3′ arm of a long-distance structure located deep within an intron 7 [259].

### 5.2. Diseases Associated with Microsatellite Mutation

Microsatellites are 2–10 bp-long repetitive DNA sequences which are abundant in human genome and, due to their structural properties, they sporadically undergo pathogenic expansions or contractions as a result of aberrant replication or DNA repair [293]. They exert distinct and position-dependent misprocessing of their host genes (e.g., transcription, splicing, nuclear export, translation) and underlie a large group of hereditary neuronal, muscular, and other diseases [294]. The extensive studies have been carried out to decipher their pathomechanism and disease hallmarks which are of great value for potential therapeutic interventions and diagnosis.

A common feature of the majority of these mutations within transcripts is their propensity to form intrinsic secondary structures of different stability and with sequence- and position-dependent functional relevance in pathomechanism of the diseases [295]. For example, their occurrence within a 5′UTR may intercept transcription (in, e.g., fragile X syndrome (FXS)) or lead to expression of toxic and prone to aggregation polyglutamine peptides (in, e.g., fragile X-associated tremor ataxia syndrome (FXTAS)) [294]. Contrarily, most intronic mutations disrupt pre-mRNA processing enabling the mutant transcript to acquire a new pathological function according to gain-of-function mechanism (in, e.g., DM) [294]. 

Myotonic dystrophy type 1 and type 2 (DM1 and DM2, respectively) constitute illustrative examples of neuromuscular diseases with an interplay between RNA secondary structure and splicing playing a crucial role in their pathomechanism. DM1 and DM2 manifestation relies on microsatellite mutations occurring either within 3′UTR of dystrophia myotonica protein kinase (*DMPK*) transcript or intron 1 of cellular nucleic acid-binding protein (*CNBP*) transcript, respectively [296,297,298]. DM1 is characterized by expansion of CUG repeats (CUG)^exp^, which impair nuclear export of its host transcript, leading to the formation of nuclear RNP inclusions [33,299,300,301]. These intramolecular inclusions, apart from toxic RNAs and as expected also other RNA species, are composed of multiple sequestered proteins including splicing factors and RNA remodelers mainly MBNL1, but also hnRNPs and RNA helicases [245,302]. These factors are either indirectly sequestered by toxic RNA or display high affinity to the RNA linear and/or structural motifs which acquire in vitro different forms of asymmetrical hairpins and brunched structures [303]. Although the DM1 molecular phenotype is a result of multiple deregulated processes, the primary one relies on reduction of functional pool of a whole family of MBNL proteins which leads to global missplicing of hundreds of alternative events [4]. These changes impair the development and function of multiple organs, especially striated muscles and brain, with the extent of severity depending on the tissue-specific expression level of a host transcript, MBNLs and the CUG repeat number [301]. Since DM1 is incurable and fatal there is an urgent need for designing effective medicaments to delay and eventually cease the progression of the disease especially life-threatening atrophy of respiratory muscles and heart failure. Many methods for screening potential medicaments have been studied on DNA, RNA, and protein levels [304,305]. The most numerous and promising group of potential therapeutics is composed of small compounds and antisense oligonucleotides which release sequestered proteins from RNP inclusions due to their high affinity or complementarity to toxic repeats and sometimes induction of degradation of toxic RNA [305,306,307,308,309]. An antisense oligonucleotide-based reagent, ISIS-DMPK-2.5_RX_, was the first potential DM1-specific drug which underwent clinical research [304,310]. However, due to low therapeutic effect in DM1 patients’ tissues the trial was halted whereas new potential reagents are being intensively screened [309,311,312,313].

Contrary to DM1, the DM2-associated CCUG expansion (CCUG)^exp^ was recently shown to promote retention of a host intron 1 (IR) [314]. The mechanism of IR is yet undefined, but it may rely on either steric hindrance of spliceosome or occlusion of *cis*-regulatory elements conveyed by structural conformation of the repeats and created RNP complexes. Interestingly the IR-based pathomechanism turned out to be relevant for other diseases with intronic GC-rich but not A/AT-rich microsatellite mutations including C9orf72-linked amyotrophic lateral sclerosis with frontotemporal dementia (C9-ALS/FTD) and Fuchs endothelial corneal dystrophy (FECD). This difference may arise from structural stability of GC-rich mutations compared to those enriched in A/ATs [295]. In addition, (CCUG)^exp^-based pathomechanism seems to differ from DM1. Although MBNLs exert higher affinity to CCUG than CUG repeats [33,172], their binding and hence sequestration in DM2 is most likely compromised by RBFOX [315]. The latter specifically recognizes a UGCAUG sequence motif [219] but also interacts with CCUG repeats as its subsidiary motif but with lower affinity. This phenomenon could explicit much milder symptoms and later onset of DM2 in contrast to DM1 [316].

### 5.3. Cancer

The range of deregulated processes in cancer cells make it impossible to emerge early molecular alterations leading the cell to a tumorigenic pathway. AS is one of these processes which is vastly disturbed and contributes to nearly all deleterious cancer cell phenotypes including metastasis, angiogenesis, or proliferation [317,318,319,320,321]. The enrichment of alterations of RNA folding in cancer genomes, whether directly imposed by riboSNitches or indirectly through mutations or alterations in expression of RNA remodelers, was found to be most likely pathogenic and could be accountable for cancer-associated molecular changes including missplicing [139,140,322]. The function of RNA structure remodelers and their structure-related effect on *cis*-regulatory elements are greatly correlated with AS regulation of proto-oncogenes and tumor suppressors [318,323]. Due to the fact that in various cancer types their level is substantially altered, the mechanism and functional relevance of RNA remodelers in pathogenic and invasive phenotype of cancer cells remain under extensive investigation [321,324]. Two studies have recently described the dysregulation of a complex network of interactions between RNA structure and RNA remodelers in esophageal squamous carcinoma cancer (ESCC) emphasizing its functional role in cancer development [139,140]. In physiological conditions, AS of a tumor suppressor pre-mRNA called receptor expressed in lymphoid tissues-like 2 (*RELL2*) is only subtly regulated by dsRNA-dependent activity of ADAR [140,325]. However, in cancer cells the level of ADAR is elevated leading to substantial exclusion of *RELL2* exon 3. In consequence, an alternative isoform prone to NMD is produced, promoting tumorigenesis. Revealed mechanism shows that ADAR binds dsRNA formed between GA-rich regions at exon 3 and an upstream Py-tract which impedes U2AF65 association with 3′ss for exon recognition [140]. Adding to the complexity of this network, DHX9 helicase which is overexpressed in different types of cancer including ESCC, was found to structurally rearrange ADAR’s RNA-substrates bidirectionally altering the downstream effect of ADAR on AS and exhibiting functional importance in tumorigenicity [139]. 

G-rich elements with a propensity to form stable RNA- and DNA-G4s, also correlate with tumorigenesis underlying many abnormalities during gene expression [326], whereas G4-targeted small compounds, stabilizing the G-quadruplexes, were found deleterious for the viability of cancer cells [327]. Recently, G-rich ISE was found crucial for AS of CD44 molecule (Indian blood group) (*CD44*) transcript due to binding hnRNP F and mediating the production of epithelial-specific *CD44* isoform. Conversely, hnRNP F depletion contributed to epithelial-to-mesenchymal transition (EMT) which associates with tumor invasive and survival properties, whereas breast cancer patients with hnRNP F lowly expressing tumors exhibited a lower survival rate [240].

## 6. Conclusions and Future Directions

Here, we provided an overview of several issues in relation to the role of RNA structural arrangement in regulation of alternative splicing in eukaryotic genes. We mainly focused on cellular modulators of RNA structure and the mechanisms undertaken by RNA structure to govern AS in physiological and pathological conditions. 

Increasing lines of evidence indicate RNA structure as a significant regulatory layer in the control of gene expression. It becomes more rational to perceive RNA structures as executors of gene-encoded information exploiting RBPs remodelers and a network of interactions to direct AS. Thus, the role of RNA conformation in AS is expected to be wide-ranging, but its experimental confirmation sometimes remains out of reach. Its complex nature including heterogeneity, dynamics as well as its interaction network with cellular environment, biomolecules and ongoing biological processes make it challenging to decipher RNA structurome and its functional relevance. The direction of future approaches has been already initiated by a few studies, bringing closer to our understanding the functional aspects of RNA structure and its dynamics relevant to a crucial context of cellular biomolecules, compartmentalization, development, and kinetics of RNA folding [50,78,80,113]. In addition, gaining deeper insight into the nature of RNA folding will enable to better understand the function of a wide range of RNA conformations. 

Large-scale technological advances, including in vitro structural probing or transcriptome-wide methods coupled with sequencing, have definitely brought deeper understanding in a global pattern of RNA conformation in cells, underscored its importance in association with biological processes and emerged particular structural elements alongside their potential regulatory function. However, detail-oriented analysis should always be appreciated as they deliver an intrinsic insight into the biological mechanism and enable to emerge single nucleotide details of great value for therapeutic interventions. This thorough knowledge on the functional structural elements within introns in alternative splicing regulation of disease-associated transcripts may vastly facilitate the development of potential therapeutics. First, targeting distant intronic regions potentially less abounding in regulatory elements could prevent from unintended deregulation of overlapping *cis*-acting motifs usually densely residing near alternative exons or within them, as it was shown for *SMN2* [253]. Second, the RNA structural elements may provide a higher number of potential targeted sites.

Eventually, future studies are envisioned to bring great discoveries of novel functions of RNA conformation which could, for example, serve as a binding platform for splicing factors to increase their local concentration and facilitate pre-mRNA processing or to temporarily and locally coordinate highly packed condensates. 

## Figures and Tables

**Table 1:** Preferences of selected RBPs towards RNA linear consensus motifs and RNA structural arrangement.

	RBPs Regulating AS	Linear Sequence Motif	RNA Structural Preferences
**1**	**CELF/BRUNOL** (CUG-binding protein Elav-like)	UGUGUGU [208]	ssRNA [209]
**2**	**FMRP** (Fragile X mental retardation protein)	G-rich elements [210]	dsRNA-rG4 [210]
**3**	**FUS** (Fused in sarcoma)	AU-rich element [211] GUGGU in a G-rich context [212]	ssRNA, stem-loop [211]
**4**	**Hu/ELAV-like** (Embryonic lethal/abnormal vision-like protein)	YUUR ^1^ interrupted by G [205] GU-rich, secondary motif AU-rich [213]	ssRNA [213]
**5**	**MATR3** (Matrin-3)	CAUCUU, AAUCUU [208]	ssRNA [167]
**6**	**MBNL** (Muscleblind-like protein)	YGCY ^2^	ssRNA, semi-stable RNA structures [172]
**7**	**NOVA** (RNA-binding protein Nova-1)	YCAY in a Y-rich context ^2^ [214]	ssRNA [214,215]
**8**	**PTBP1** (Polypyrimidine tract-binding protein 1)	YUCY [205], YCTY, YGCY ^2^ [38]	ssRNA, Internal loop [216]
**9**	**PUF60** (Poly-U-binding factor 60 kDa)	U-rich elements [217]	ssRNA [217]
**10**	**QKI** (Quaking STAR protein)	ACUAAC, NACUAAY-N_1-20_-UAAY ^2,3^ [218]	ssRNA, hairpin loop [215]
**11**	**RBFOX** (RNA binding protein fox)	UGCAUG [208]	ssRNA [219], stem-loop [220]
**12**	**RBM4** (RNA binding protein 4)	CGGG [221]	ssRNA, stem-loop structure [222]
**13**	**RBMY** (RNA-binding motif protein, Y chromosome)	CA/UCAA [223]	ssRNA, stem-loop [223]
**14**	**SAM68** (Src-Associated substrate in Mitosis of 68 kDa)	UAAA, UUAA, U-rich [224]	ssRNA, internal/hairpin loop [224]
**15**	**STAU1** (Double-stranded RNA-binding protein Staufen homolog 1)	none	dsRNA [225]
**16**	**TAF15** (TATA-box binding protein Associated Factor 15)	GGUAAGU [226], GGUG [227]	ssRNA, hairpin loop [227]
**17**	**TDP-43** (TAR DNA-binding protein 43)	RUGY ^1,2^ [205]	ssRNA [228]
**18**	**TIA1** (T-cell intracellular antigen 1)	AU-rich elements [229], TTTA [205], UUUUUUC/A [206]	ssRNA [230], stem-loop [231]
**19**	**TIAL1** (T-cell intracellular antigen 1-like 1)	AU-rich elements [229], Poly(U) [230]	ssRNA [230]

^1^ R, A or G; ^2^ Y, C or U; ^3^ N, any base.
